# Location of Nociceptive Stimulus in the Peripersonal Space Impacts Pain Experience

**DOI:** 10.1111/psyp.70396

**Published:** 2026-09-05

**Authors:** Béla Bucsai, Áron Horváth, Ádám Koncz, Ferenc Köteles

**Affiliations:** ^1^ Institute of Psychology ELTE Eötvös Loránd University Budapest Hungary; ^2^ Ádám György Psychophysiology Research Group Budapest Hungary; ^3^ Institute of Psychology Károli Gáspár University of the Reformed Church in Hungary Budapest Hungary; ^4^ Institute of Health Promotion and Sport Sciences ELTE Eötvös Loránd University Budapest Hungary

## Abstract

Peripersonal space (PPS) is the area surrounding the body where we can reach, see, and interact with objects, acting as a dynamic interface between us and the environment. We expected that pain stimuli closer to the body center in the PPS would result in higher pain intensity, increment in heart rate (HR), and skin‐conductance response (SCR). It was also assumed that proprioceptive systematic error toward the body center, and psychological factors such as negative affect, pain catastrophizing, and somatosensory amplification would show a positive association with pain. Sixty individuals participated in this preregistered experiment (age: 22.9 ± 6.2 years, 76% female). Thermal pain stimulation was applied on the forearm at different stimulation sites and at different elbow joint positions. Joint Position Reproduction test in the elbow joint was used to assess proprioceptive systematic error. Psychological constructs were measured with questionnaires. Based on linear mixed models, elbow position, and stimulation site showed significant main effects and interaction when the outcome was pain intensity. By adding psychological factors to the models as covariates, pain catastrophizing showed a positive association, while state negative affect showed a negative association with perceived pain. Trait negative affect, somatosensory amplification, and proprioceptive accuracy did not influence pain reports. Beyond the traditionally included tactile, visual, and auditory modalities, proprioception should be considered in a more complex conceptualization of PPS. Our results indicate that the location of thermal stimulation might influence pain intensity within the PPS, and that a complex interplay exists between proprioceptive information and stimulation site.

## Impact Statement

Our experiment tested the idea that the position of nociceptive stimuli in the peripersonal space (PPS) can modify the intensity of pain, even when participants rely on proprioceptive information (closed eye) about the position of the stimulus. Our findings suggest the existence of a center‐to‐periphery gradient within the PPS and propose that proprioception should be taken into consideration in a more complex conceptualization of PPS.

## 1 Introduction

### 1.1 Peripersonal Space (PPS) and Pain

The existence of a map that surrounds the body, relying on neurons responsive to both visual and somatosensory information, was first shown in macaque monkeys (Graziano and Gross 1992, 1993; Rizzolatti et al. 1981). The mapped area, called peripersonal space (PPS), is the space where interactions between the self and the environment take place (Bogdanova et al. 2021; de Vignemont et al. 2021). Therefore, the PPS is assumed to play a pivotal role in the organization of visually guided actions, including not only the desired contacts between external objects and the body but also the avoidance of unwanted physical interactions (Koppel et al. 2023; Noel et al. 2021; Sambo and Iannetti 2013). Indeed, it is hypothesized that a defensive sensorimotor (body schema) representation of the body, that is, a margin of safety, also exists (Finisguerra et al. 2015; Graziano and Cooke 2006; Makin et al. 2009). This representation “maps out parts of our bodies that we should pay special attention to” (Klein 2015, 94). Based on these findings and ideas, de Vignemont proposed the so‐called *bodyguard hypothesis*, stating that the experience of body parts is incorporated in a protective body schema (de Vignemont 2017, 2018). Enhanced vigilance and preparedness primarily refer to external factors with an assumed negative effect on the integrity of the body.

Pain is considered a homeostatic emotion with the ultimate function of protecting the body from actual and expected damage (Craig 2003; IASP 1994; Raja et al. 2020). Thus, there is an obvious overlap between the defensive aspect of the PPS and the primary function of pain; both phenomena were developed to avoid bodily damage in a predictive way. Although pain experience typically relies on the activation of nociceptors located in many tissues of the body, it is well‐known that it is substantially modulated by top‐down factors, such as context, emotional state, and expectations (Benedetti 2009; Meagher et al. 2001; Rhudy et al. 2008; Villemure and Bushnell 2002). Considering the functional overlap between pain and the PPS, it can be assumed that if the potentially painful stimulus is localized in the PPS, the latter will enhance the salience of pain (De Paepe et al. 2014), allocating more cognitive capacity for its processing (Legrain et al. 2011). If the stimulus actually elicits pain, a more thorough processing can intensify the pain experience, thus facilitating the defensive response. To the authors' knowledge, however, this idea has never been empirically tested to date.

Beyond the experience of pain, acute painful stimulation increases the activity of the sympathetic nervous system, leading to various peripheral changes, such as increased heart rate (HR), blood pressure, and skin‐conductance response (SCR) (Boucsein 2012; Burton et al. 2009; Delity et al. 2023).

### 1.2 Heterogenety of the PPS

The PPS has heterogeneous effects on the salience of potentially or actually threatening stimuli (Noel et al. 2021). For example, blink reflex elicited by the electrical stimulation of the wrist was more pronounced when the hand was in the proximity of the face (De Paepe et al. 2016; Sambo, Forster, et al. 2012; Sambo, Liang, et al. 2012). Also, cue‐pain associations with respect to the face are learned more readily than to the hand (Schmidt et al. 2020), and in patients with trigeminal neuralgia, a disease characterized by unilateral pain, PPS was enlarged around the impacted side of the face (Bufacchi et al. 2017). In line with these results, findings of an empirical study suggest that there is an “ultra‐near” defensive space in the close proximity of the face, and the extent of that space is positively associated with trait anxiety (Sambo and Iannetti 2013).

Beyond these major differences, it is not clear whether there is a gradient in pain perception within the PPS. In order to study this idea further, one has to determine the possible directions along which such a gradient could exist. The frontal midline of the body is considered an important egocentric reference frame (Ball et al. 2010); the area of the PPS around the frontal midline can be characterized by a higher level of attention, perhaps because this is where manual manipulation of objects takes place and/or there are crucially important and sensitive internal organs in the chest (Gács 2019). In empirical studies, more attention was allocated to stimuli presented closer to the body (i.e., within the PPS along the sagittal axis) than beyond the PPS (Couyoumdjian et al. 2003; de Gonzaga Gawryszewski et al. 1987; Losier and Klein 2004; Plewan and Rinkenauer 2017). It can also be assumed, partly based on the existence of a proximal‐to‐distal gradient in nociceptor afferent density in the limbs (McArthur et al. 1998), that nociceptive stimuli administered more proximal to the trunk evoke more pain than more distally located stimuli.

### 1.3 Proprioception and the PPS

It has been proposed that the PPS is the product of integration of information from the tactile and visual or auditory modality (Blini et al. 2021; Noel et al. 2021). However, for stimulation that actually reaches the body, such as tactile and nociceptive stimuli, there is another potential source of information about the position of the stimulus in the PPS, namely the proprioceptive modality. As the bodyguard hypothesis and other similar ideas conceptualize the defensive aspect of PPS as a sensorimotor representation (de Vignemont 2018; de Vignemont et al. 2021; Finisguerra et al. 2015; Makin et al. 2009), it is likely that proprioceptive information also contributes to this representation (Dijkerman and Medendorp 2021; Reed and Park 2021). Thus, it is possible that even in the absence of visual information (e.g., with closed eyes), the intensity of pain evoked by a nociceptive stimulus is modulated by proprioceptive information with respect to its actual position. To the authors' knowledge, this idea has been investigated only in a doctoral work (Gács 2019) in comparatively small samples (*n* = 20) to date. According to the results of three experiments, thermal pain delivered to the dorsal surface of the forearm was consistently significantly more intense when the forearm was closer to the frontal plane of the body. Concerning the median sagittal plane (midline), the difference between medially and laterally positioned forearm nociceptive stimulation depended on the visibility of the arm and on the distance of the lateral position from the body.

**FIGURE 1:**
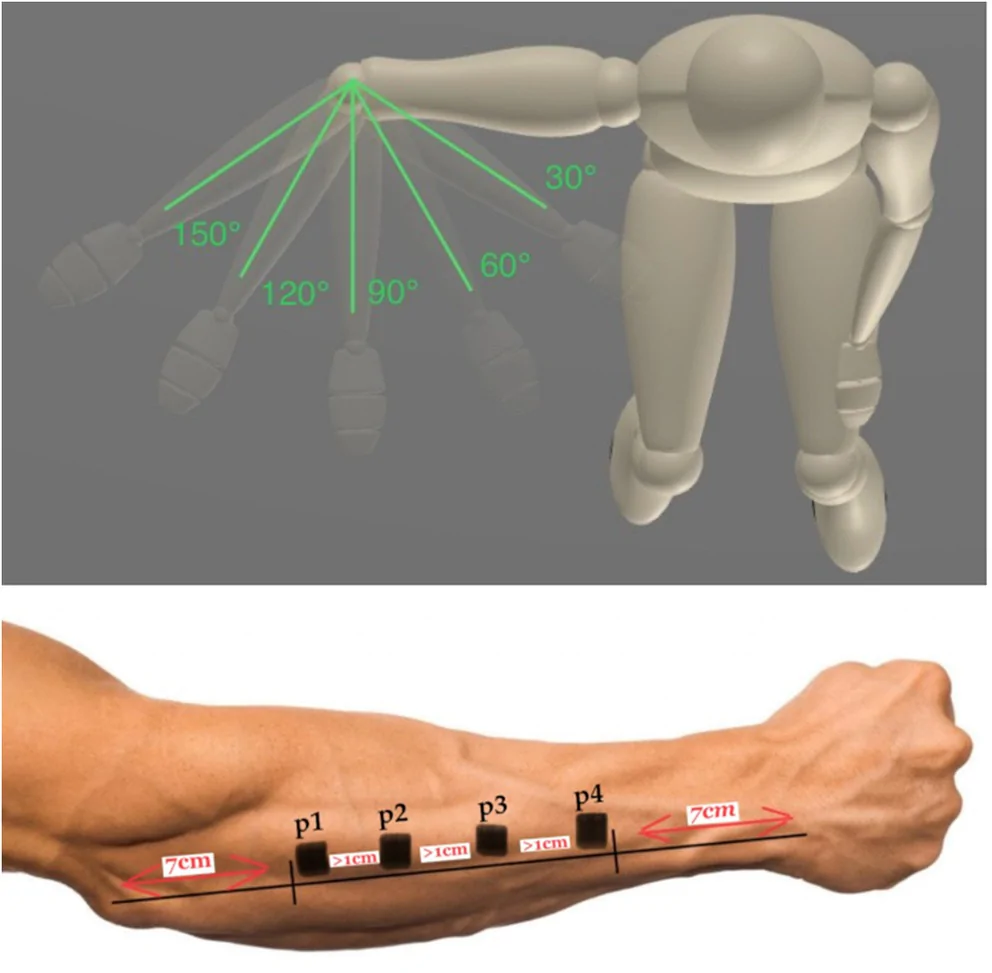
Elbow joint positions (upper part) and stimulation sites (p1–p4) along the forearm (lower part) used in the experiment.

In the study reported in the present paper, the existence of a potential gradient in the PPS was investigated through thermal pain administered to the dorsal surface of the forearm. Position of the painful stimulus within the PPS was manipulated systematically along two independent factors. First, the angle of the elbow joint was changed in 5 steps, from 30° (flexion) to 150° (extension), that is, toward the periphery of the PPS (Figure 1). Second, painful stimulation was delivered on four points along the longitudinal axis of the forearm (Figure 1).

There are well‐known individual differences in the accuracy of perception of the actual angle of the joints (Goble 2010; Han et al. 2016; Horváth et al. 2023). These differences often include a systematic bias in the perception of the position of the joints, that is, certain individuals consistently underestimate the actual angle, whereas others tend to overestimate it (bias toward flexion and extension, respectively) (Boisgontier et al. 2012; Goble et al. 2012; Horváth et al. 2023). A further assumption of the present study was that a bias toward flexion with respect to the elbow joint, that is, when the perceived position of the forearm is closer to the midline of the body than the actual, is associated with a higher level of pain.

### 1.4 Psychological Characteristics Contributing to Pain

Beyond localization relative to the body, including proprioceptive and possibly other biases, empirical findings suggest that experienced painfulness of a nociceptive stimulus may also be impacted by various trait‐like characteristics. For example, negative affectivity (NA) (Gaskin et al. 1992; Janssen 2002), somatosensory amplification (SSA) (Ak et al. 2004; Kirk and Jahoda 2009; Kröner‐Herwig and Maas 2013; Meana and Lykins 2009; Raphael et al. 2000), and pain catastrophizing (PC) (Quartana et al. 2009; Sullivan and Tripp 2024) showed a positive association with pain in a number of studies. Taking into consideration the meaning of these constructs, these associations are well‐explainable. NA refers to the disposition to experience aversive emotional states (Watson and Clark 1984), SSA was recently reconceptualized as amplification of somatic threats (Köteles and Witthöft 2017), and PC is an exaggerated negative mental response to pain (Thompson et al. 2022). In summary, these trait‐like psychological characteristics can be considered top‐down factors contributing to the response to pain.

### 1.5 Hypotheses of the Study

In the experimental study reported in the present paper, the following hypotheses were preregistered and tested. First, there is a negative association between pain intensity in the forearm and the angle of the elbow joint (H1). Beyond pain intensity, a self‐reported variable, the angle of the elbow joint was assumed to show a negative association with SCR (H3) and HR (H4). Second, more distal stimulation on the forearm was expected to be associated with less intense pain (H2). The fifth hypothesis (H5) is that systematic error in proprioceptive accuracy of the elbow joint is associated with pain intensity, that is, distortions toward the body are associated with more intense pain. Finally, we also expected somatosensory amplification (H6), negative affect (H7), and pain catastrophizing (H8) to be positively associated with pain intensity.

## 2 Method

### 2.1 Study Approval

The study was approved by the Research Ethics Committee of the Faculty of Education and Psychology at ELTE Eötvös Loránd University under the registration number 2024/393. The study was preregistered at https://osf.io/sqcuj (Bucsai et al. 2025). The data are openly available at: https://osf.io/8x9y7/?view_only=a4ed5c09f49a4ffcbc954af87118f5c7.

### 2.2 Participants

To determine the optimal sample size a priori power analysis was performed using G*Power software (version 3.1.9.7) (Erdfelder et al. 1996) for the planned four‐level repeated measures ANOVA, with α = 0.05, 1−β = 0.8 and medium effect size (*f* = 0.25). The analysis resulted in a minimum of 60 participants. The required sample size for repeated measures ANOVA is a good approximation of the sample size requirements for Linear Mixed Models (LMM) applied in the analysis of the present data (Guo et al. 2013).

Among the inclusion criteria, participants must have had intact (uninjured) skin on the stimulated area and must not have any medical condition/treatment that could alter or influence pain perception. In total, we obtained data of 70 participants. Due to low pain intensity scores (early habituation to stimuli) and an extensive amount of missing data (participants forgot to rate pain sometimes), we removed data of 10 participants from the analyses. The final number of participants included in the main analyses was 60. Their mean age was 22.9 years (SD = 6.15) and 76% was female.

Due to the malfunction of one of the NeXus devices, we lost the SCR data of 13 and the HR data of 16 participants. As a result of internet failures during the early stages of the study, we lost some questionnaire and proprioceptive accuracy data; the final number of included participants in the related analyses is shown in Table 1.

**TABLE 1:** Descriptive statistics of the variables.

Variable	Valid	Missing	Mean	SD	Min	Max
Pain intensity	60	0	29.53	23.86	0	97
SCR (μS)	47	13	0.35	0.38	0	2.62
HR change (bpm)	44	16	−2.58	5.42	−24.27	19.54
PSE 30°	51	9	−0.25	5.65	−10.23	16.83
PSE 60°	51	9	−1.59	3.22	−9.83	5.83
PSE 90°	51	9	−0.15	3.02	−9.20	6.43
PSE 120°	51	9	2.17	3.21	−5.38	8.20
PSE 150°	51	9	3.04	6.17	−8.40	24.50

Abbreviations: HR, heart rate; PSE, roprioceptive systematic error; SCR, skin‐conductance response.

### 2.3 Psychological Measures

#### 2.3.1 Pain Intensity

Pain intensity was assessed with a visual analogue scale (VAS) presented digitally (via a tablet device). The scale consisted of a 17 cm bar transitioning from green to red, on which participants marked the pain intensity after each stimulation on a scale from 0 to 100. Below the scale, the values were labeled as 0 (“no pain”), 50 as a central reference point, and 100 (“strongest imaginable pain”).

#### 2.3.2 Negative Affect

The Positive and Negative Affect Schedule (PANAS) (Watson et al. 1988) is a 20‐item self‐report scale designed to measure positive and negative affect; but in this study, only those 10 items assessing negative affect, that is, the propensity to experience negative affective states, were used. Depending on the instructions, the questionnaire can measure either trait or state affect; in the present study, both types of instructions were used. Items are rated on a 5‐point Likert scale ranging from “not at all” or “very slightly” to “extremely”. The Hungarian version of the questionnaire was found to be valid and psychometrically sound (Gyollai et al. 2011). The internal consistency was acceptable in the present study, with Cronbach's α being 0.73 for the state, and 0.71 for the trait negative affect.

#### 2.3.3 Somatosensory Amplification

The Somatosensory Amplification Scale (SSAS) (Barsky et al. 1990) is a 10‐item questionnaire that measures the tendency to perceive somatic sensations as harmful, intense, or noxious. Respondents are asked to rate items on a 5‐point Likert scale ranging from “not at all” to “very much”. Higher scores mean a greater tendency toward amplification. The Hungarian version of the questionnaire proved to be valid and psychometrically sound (Köteles et al. 2009). In the present study the internal consistency was just below, but near the acceptable level (Cronbach's α = 0.64).

#### 2.3.4 Pain Catastrophizing

The Pain Catastrophizing Scale (PCS) (Sullivan et al. 1995) is a 13‐item self‐report questionnaire, assessing the exaggerated negative orientation toward noxious stimuli, like rumination, magnification and helplessness. Items are rated on a 5‐point Likert scale ranging from 0 (“not characteristic at all”) to 4 (“completely characteristic”). A higher score indicates a greater tendency toward catastrophizing. The Hungarian version of the questionnaire was found to be valid and psychometrically appropriate (Kökönyei 2008). In the present study Cronbach's α was 0.87.

### 2.4 Physiological Measurements

The NeXus system (NeXus‐10 Mark II, Version 1.02 device and BioTrace + Software for NeXus‐10 v2018A1; Mind Media BV, Herten, the Netherlands) was used for the assessment of physiological changes. Electrocardiography (ECG) was recorded using the modified Lead II electrode arrangement (right clavicula and the lower part of the left ribcage). Skintact Ag/AgCl ECG electrodes were used, and the electrical signal sampling frequency was 1024 Hz with a 50 Hz notch filter. SCR was measured with electrodes placed on the middle phalanges of the middle and index fingers of the right hand on the palmar side, and electrical signal sampling frequency was 128 Hz.

When recording the responses, we put a marker in the data before each trial. The data were prepared for analysis in a Python 3 runtime environment using the google colab software. The mne (Gramfort et al. 2013) library was used to manipulate the EDF files, and the pandas (The Pandas Development Team 2024) and numpy (Harris et al. 2020) libraries were used to perform operations on the data. For the analyses, segments of 10 s were cut from the data, around the marker (baseline) and from the start of stimulation (baseline +14 s). For the SCR, we calculated the minimum values of the baseline segments and the maximum values of the stimulation segments, and their signed difference was used in the analyses (higher values indicate a greater change in SCR). For the HR data, we calculated the average of the baseline and the average of the stimulation segments, and their signed difference was used in the analyses (higher values indicate an elevation of HR).

### 2.5 Measurement of Proprioceptive Systematic Error

The Joint Position Reproduction (JPR) test was used to assess proprioceptive systematic error. A custom‐made motorized device, with a precision of ±0.1 degrees, controlled and measured the position of the right elbow joint during the measurement. Participants' eyes were covered during the assessment. The joint was passively moved with a predetermined speed to one of the five starting positions (30°, 60°, 90°, 120°, 150°, see Figure 1) and then the joint was moved to one of the target positions (30°, 60°, 90°, 120°, 150°) where it remained for 6 s. After the waiting period, the forearm was moved back to the starting position and then directed toward the target position again. Participants had to indicate when their arm reached the target position by pressing a button. Starting and target positions were indicated by different auditory signals, and all possible combinations of the five starting and target positions were presented, resulting in a total of 20 trials which were administered in a randomized order (trials with identical starting and target position were excluded). To evaluate participants' performance, we used the systematic error score, calculated as the signed mean difference between the target and reproduced positions. A negative value means that the distortion occurs toward the body's center, while a positive value indicates a distortion in the direction away from the body. As proprioceptive systematic error might be joint position specific (Horváth et al. 2023), it was calculated separately for the five possible joint positions.

### 2.6 Thermal Stimulation

The heat stimulation device (MHC V4.1, LRG Ltd., Győr, Hungary) was equipped with four independently operable stimulation heads with a Peltier thermoelectric module, which were attached to the participant's right forearm. The heads, following the procedure of Gács (2019), were placed on the dorsal surface of the right forearm on the C7 dermatome, along a line between the caput ulnae and the lateral epicondyle, at equal distances from each other and 7 cm from both endpoints (Figure 1). A minimum distance of 1 cm was maintained between the heads to prevent spatial summation.

Before the start of the experiment, each participant was presented with the heat stimulus at the stimulation site closest to the wrist. Each stimulation was preceded by a preheating phase, during which the temperature of the heads was kept at 35°C for 5 s. Afterwards they were heated up to the target temperature of 43.5°C at a rate of 2.5°C/s in 3.4 s and remained at this temperature for 5 s. This temperature stimulates the nociceptors in the skin but does not cause tissue damage (Martin and Falder 2017). This procedure ensured that all participants experienced the same temperature difference, regardless of external conditions such as room temperature, skin temperature, or the initial temperature of the stimulating heads.

### 2.7 Procedure

Participants were measured one by one in one session. Upon arrival at the laboratory, they read and signed the informed consent form (they received the information that the study investigates the associations between arm position and pain; no further details on the hypotheses were provided) and completed the questionnaires. Afterwards, the electrodes were mounted, and participants completed the JPR, followed by the pain stimulation task. In the stimulation task, the elbow joint angle was set with the motorized device used to measure proprioceptive accuracy (see above). There were five different elbow joint angles (30°, 60°, 90°, 120°, 150°) and four stimulation sites (from s1 to s4, with s1 being the most proximal to the elbow joint, and s4 the most distal; see Figure 1); in order to prevent temporal summation, for each elbow joint position–stimulation site combination three heat stimuli were presented in a quasi‐random order, ensuring that the same combination did not occur consecutively. Thus, participants received a total of 60 nociceptive stimulations. After each stimulation, participants evaluated pain intensity using a visual analogue scale (VAS). Participants' eyes were covered during the assessment.

### 2.8 Statistical Analysis

To ensure there are no artifacts, during the data collection we noted every unusual event that could lead to elevated sympathetic activation, such as loud noises from the environment, objects falling to the ground, or someone interrupting the experiment. In order to identify recording artifacts, we visually examined all the physiological data (HR and SCR), looking for unusual patterns. After identification, we manually removed all the artifacts from the dataset. Also, we identified outlier values using the Interquartile Range (IQR), defined as data points falling below Q1–2.5 * IQR or above Q3 + 2.5 * IQR. All identified data points were visually inspected to confirm their status as genuine anomalies rather than valid extreme values. Following this verification, confirmed artifacts were removed from the dataset prior to subsequent analysis.

To test the expected effects of elbow joint position (H1) and stimulation site (H2), an LMM was conducted. Elbow joint position (5 levels), stimulation site (4 levels), and log transformed version of trial number (to control for habituation; for details, see the Supporting Information) were added to the model as fixed effects. As a random effect Participant ID was added to account for the repeated measures, and log transformed version of trial number was added to account for the individual differences in habituation. After that, to test the possible role of psychological factors (H6‐8), SSAS, PCS, and PANAS state and trait version were separately added to the model as covariates. To test H5, linear regression models were conducted, where the outcome variable was pain intensity in a given join position, and the predictor was proprioceptive systematic error in the joint position at hand. In the preregistration we claimed to use ANCOVA, but later decided that this analysis might be a better solution to reflect the joint‐position‐specific nature of proprioceptive error (Horváth et al. 2023). To test the effect on physiological variables (H3 and H4), the same models were conducted with SCR and HR as outcome variables, and without covariates. For the post hoc comparisons, we used Holm‐corrected *p*‐values with the standard 0.05 significance level.

## 3 Results

### 3.1 Pain Intensity, Elbow Joint Angle (H1) and Stimulation Site (H2)

Descriptive statistics are presented in Table 1. An LMM was fitted (χ^2^(23) = 3221.39, *p* < 0.001, Conditional *R* ^2^ = 0.640), explaining 64% of the total variance with the fixed effects explaining 4.3% of the variance (χ^2^(20) = 206.867, *p* < 0.001, Marginal *R* ^2^ = 0.043). The ANOVA Fixed Effect Omnibus tests showed a significant elbow joint angle main effect with a very small effect size (F(4, 3393) = 6.72, *p* < 0.001, ηp^2^ = 0.008). The stimulation site also showed a significant main effect with a very small effect size (F(3, 3384) = 3.53, *p* < 0.001, ηp^2^ = 0.003). Trial number showed a significant main effect with a large effect size (F(1, 64.7) = 55.19, *p* < 0.001, ηp^2^ = 0.460) displaying a logarithmic fit (see Figure S1). The elbow joint position * stimulation site interaction showed a significant effect with a small effect size (F(12, 3393) = 10.66, *p* < 0.001, ηp^2^ = 0.036).

**FIGURE 2:**
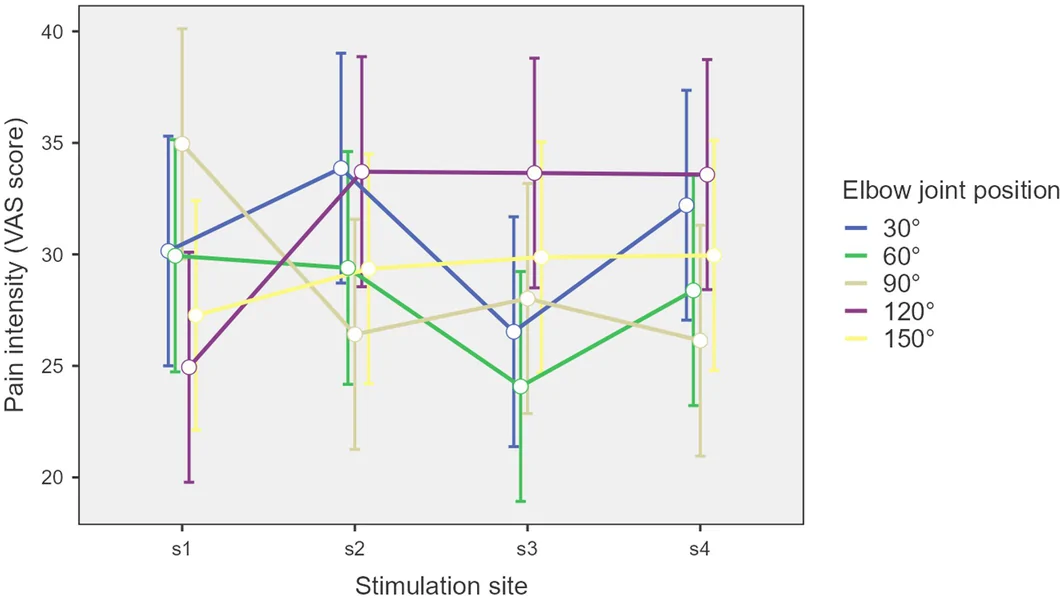
Pain intensity per elbow joint position and stimulation site (error bars represent 95% confidence intervals).

To understand the interaction, we compared the pain intensity for the elbow joint positions at different stimulation sites (See Figure 2 and Table 2).

**TABLE 2:** Pain intensity (VAS) per elbow joint position and stimulation site (estimated marginal means ± SE).

Elbow joint position	Stimulation site			
	s1	s2	s3	s4
30°	30.2 ± 2.59	33.9 ± 2.59	26.5 ± 2.59	32.2 ± 2.59
60°	29.9 ± 2.62	29.4 ± 2.63	24.1 ± 2.59	28.4 ± 2.60
90°	35.0 ± 2.60	26.4 ± 2.59	28.0 ± 2.59	26.1 ± 2.60
120°	24.9 ± 2.59	33.7 ± 2.59	33.6 ± 2.59	33.6 ± 2.59
150°	27.3 ± 2.59	29.4 ± 2.59	29.9 ± 2.60	30.0 ± 2.59

For s1, 90° showed higher pain intensity than 120° and 150°. For s2, 90° showed lower pain intensities than 30° and 120°. For s3, 120° showed higher pain intensities than 30°, 60°, and 90° while 150° showed higher pain intensities than 60°. For s4, 90° showed lower pain intensities than 30° and 120°. In summary, most differences are due to 90° and 120° showing a different pattern across stimulation sites than other angles.

For 30°, s3 showed lower intensities from s2 and s4. For 60°, s1 showed higher pain intensity than s3. For 90°, s1 showed higher pain intensities than all the other stimulation sites. For 120°, s1 showed lower pain intensities than all the other stimulation sites. For 150°, stimulation sites showed no significant difference in pain intensity. In summary, most differences arise from s1 showing a different pattern across angles than the other stimulation sites (See Figure 2).

### 3.2 Proprioceptive Systematic Error (H5)

No significant association between proprioceptive systematic error and pain intensity was revealed in any model (*p* > 0.05) (Table 3).

**TABLE 3:** Results of regression analyses to test the effect of proprioceptive systematic error on pain intensity.

Elbow joint position	Regression model fit
30°	F(1) = 0.005, *p* = 0.942, *R* ^2^ = 0.000
60°	F(1) = 1.426, *p* = 0.238, *R* ^2^ = 0.029
90°	F(1) = 2.700, *p* = 0.107, *R* ^2^ = 0.052
120°	F(1) = 1.982, *p* = 0.165, *R* ^2^ = 0.039
150°	F(1) = 1.648, *p* = 0.205, *R* ^2^ = 0.033

### 3.3 Physiological Variables: SCR And HR (H3, H4)

**FIGURE 3:**
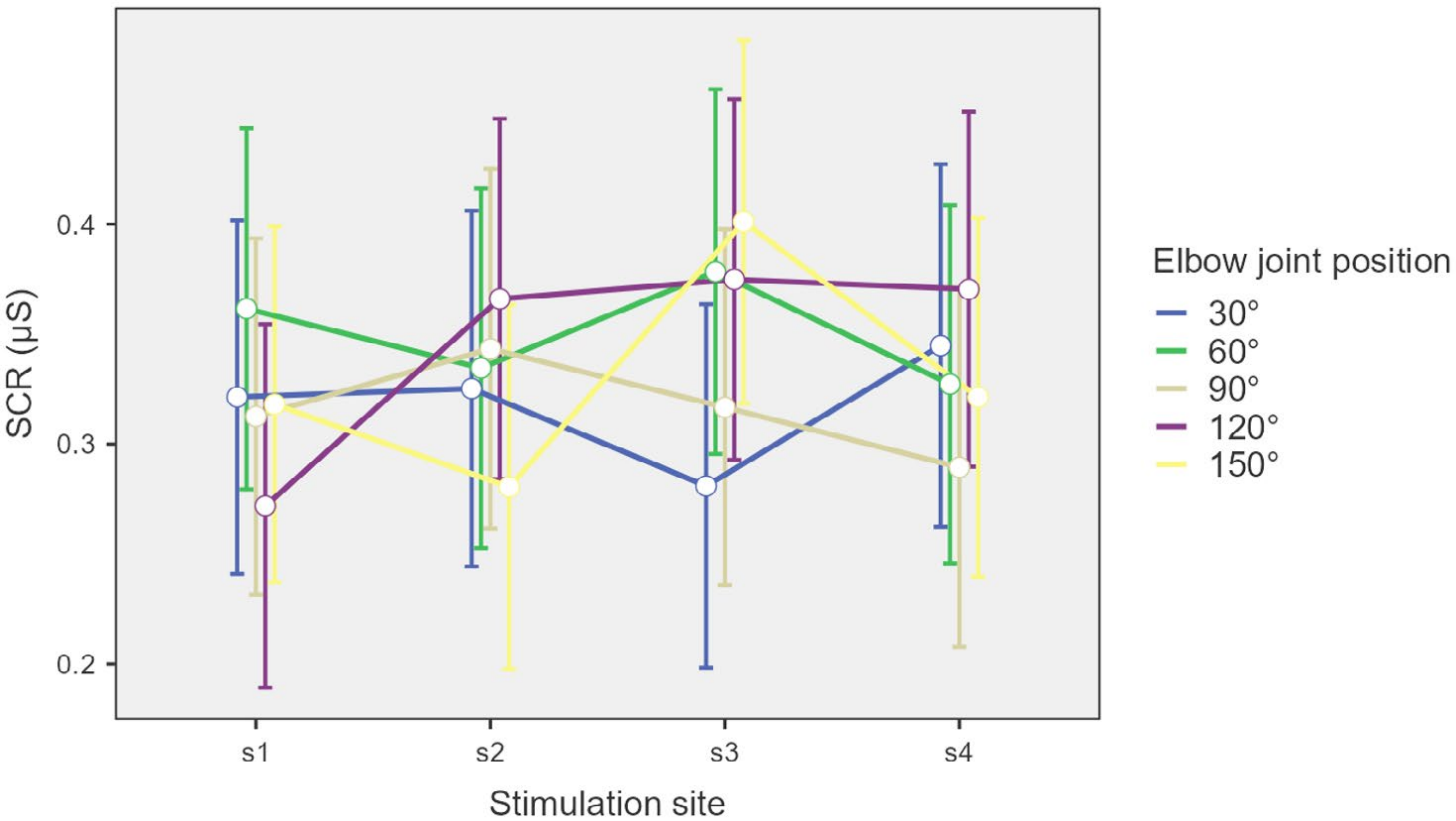
Skin conductance response (SCR) per elbow joint position and stimulation site (error bars represent 95% confidence intervals).

For SCR, an LMM was fitted (χ^2^(23) = 1278.307, *p* < 0.001, Conditional *R* ^2^ = 0.499), explaining 50% of the total variance, with the fixed effects explaining 9.5% of the variance (χ^2^(20) = 64.933, *p* < 0.001, Marginal *R* ^2^ = 0.095). The ANOVA Fixed Effect Omnibus tests showed a significant trial number main effect, with a large effect size (F(1, 50.1) = 46.29, *p* < 0.001, ηp^2^ = 0.480) while elbow joint position (F(4, 2013.7) = 1.44, *p* = 0.219, ηp^2^ = 0.003) and stimulation site (F(3, 2009.3) = 1.35, *p* = 0.258, ηp^2^ = 0.002) main effects were not significant. However, the elbow joint position * stimulation site interaction was significant, with a small effect size (F(12, 2014.7) = 1.98, *p* = 0.022, ηp^2^ = 0.012), but the post hoc tests showed no significant pairwise differences (See Figure 3 and Table 4). Overall, no interpretable effect emerged.

**TABLE 4:** Skin conductance response (μS) per elbow joint position and stimulation site (estimated marginal means ± SE).

Elbow joint position	Stimulation sites			
	s1	s2	s3	s4
30°	0.32 ± 0.04	0.33 ± 0.04	0.28 ± 0.04	0.35 ± 0.04
60°	0.36 ± 0.04	0.34 ± 0.04	0.38 ± 0.04	0.33 ± 0.04
90°	0.31 ± 0.04	0.34 ± 0.04	0.32 ± 0.04	0.29 ± 0.04
120°	0.27 ± 0.04	0.37 ± 0.04	0.38 ± 0.04	0.37 ± 0.04
150°	0.32 ± 0.04	0.28 ± 0.04	0.40 ± 0.04	0.32 ± 0.04

**FIGURE 4:**
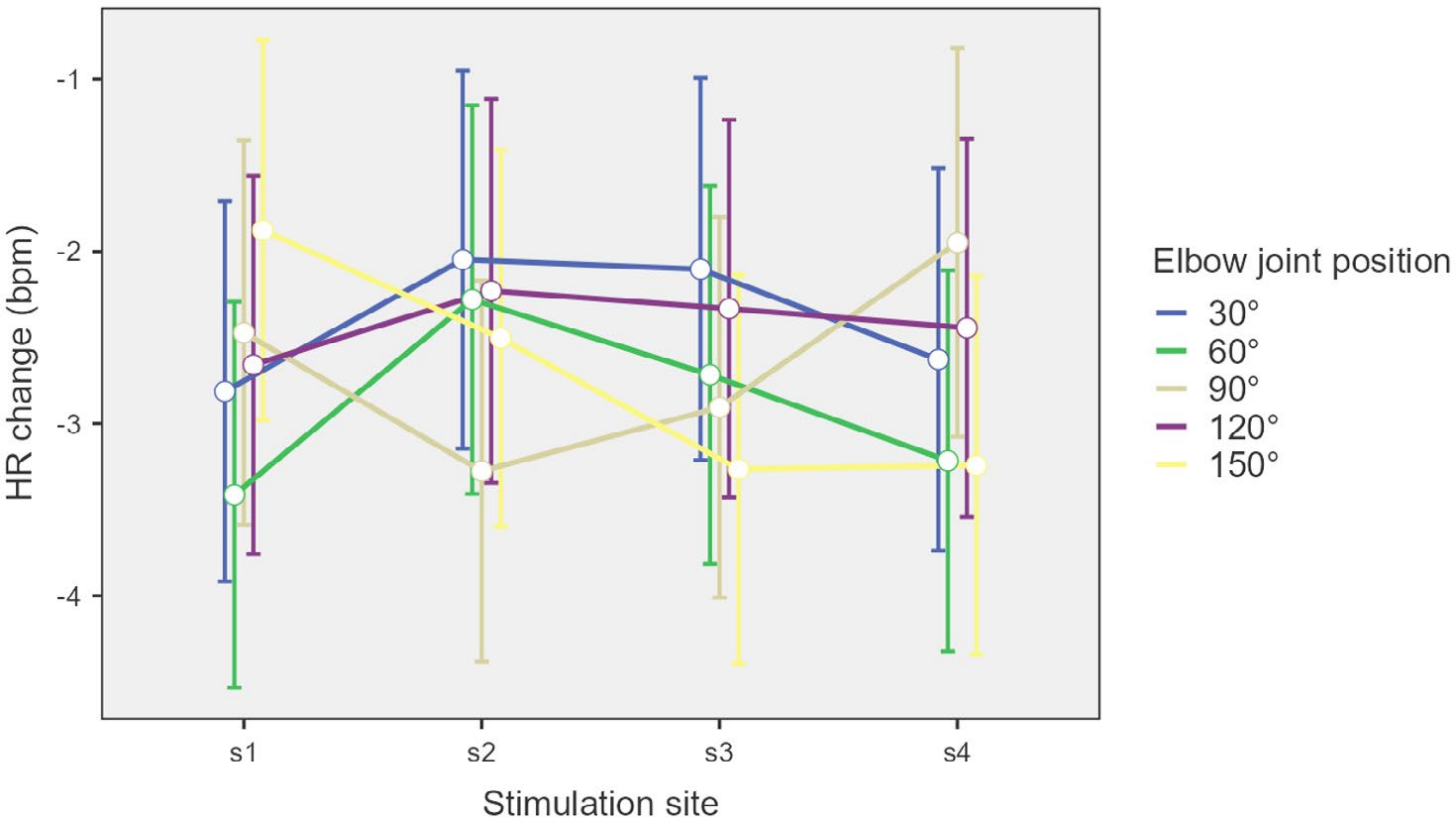
Heart rate (HR) change per elbow joint position and stimulation site (error bars represent 95% confidence intervals).

For HR, an LMM was fitted (χ^2^(23) = 387.802, *p* < 0.001, Conditional *R* ^2^ = 0.192) explaining 19.2% of the total variance but fixed effects did not explain a significant amount of the variance (χ^2^(20) = 21.983, *p* = 0.341, Marginal R^2^ = 0.007). The ANOVA Fixed Effect Omnibus tests showed that neither elbow joint position (F(4, 2384) = 0.93, *p* = 0.445, ηp^2^ = 0.002), nor stimulation site (F(3, 2370) = 0.27, *p* = 0.846, ηp^2^ = 0.000), or trial number (F(1, 50.9) = 0.00, *p* = 0.971, ηp^2^ = 0.000) had a significant main effect. The elbow joint * stimulation site interaction was also not significant (F(12, 2385.4) = 1.39, *p* = 0.163, ηp^2^ = 0.007) (See Figure 4 and Table 5). Overall, no significant effect emerged.

**TABLE 5:** Heart rate change (bpm) per elbow joint position and stimulation site (estimated marginal means ± SE).

Elbow joint position	Stimulation site			
	s1	s2	s3	s4
30°	−2.81 ± 0.56	−2.05 ± 0.56	−2.10 ± 0.56	−2.63 ± 0.56
60°	−3.41 ± 0.57	−2.28 ± 0.57	−2.72 ± 0.56	−3.22 ± 0.56
90°	−2.47 ± 0.57	−3.28 ± 0.56	−2.91 ± 0.56	−1.95 ± 0.57
120°	−2.66 ± 0.56	−2.23 ± 0.57	−2.33 ± 0.56	−2.44 ± 0.56
150°	−1.88 ± 0.56	−2.50 ± 0.56	−3.26 ± 0.57	−3.24 ± 0.56

### 3.4 Psychological Factors and Pain Intensity

Descriptive statistics of the assessed variables are presented in Table 6.

**TABLE 6:** Descriptive statistics of the psychological variables.

Variable	Valid	Missing	Mean	SD	Min	Max
PANAS negative state	58	2	30.97	6.67	15.00	43.00
PANAS negative trait	56	4	33.86	6.13	14.00	45.00
PCS	58	2	26.72	5.52	16.00	39.00
SSAS	42	18	15.17	7.80	3.00	33.00

Abbreviations: PANAS, Positive and Negative Affect Schedule; PCS, Pain Catastrophizing Scale; SSAS, Somatosensory Amplification Scale.

#### 3.4.1 Trait Negative Affect (H7)

By adding trait NA to the LMM, it explained 65.2% of the total variance (χ^2^(24) = 3083.114, *p* < 0.001, Conditional *R* ^2^ = 0.652) with the fixed effects explaining 6.7% of the variance (χ^2^(21) = 200.250, *p* < 0.001, Marginal *R* ^2^ = 0.067.), 2.4% more than in the original model. The ANOVA Fixed Effect Omnibus tests showed that elbow joint position (F(4, 3169.2) = 6.33, *p* < 0.001, ηp^2^ = 0.008), stimulation site (F(3, 3159.8) = 4.20, *p* = 0.006, ηp^2^ = 0.004), and trial number (F(1, 60.4) = 52.28, *p* < 0.001, ηp^2^ = 0.464) remained significant while trait NA (F(1, 54) = 2.58, *p* = 0.114, ηp^2^ = 0.046) showed no significant effect. The elbow joint position * stimulation site interaction (F(12, 3169.4) = 10.07, *p* < 0.001, ηp^2^ = 0.037) remained significant as well.

In summary controlling for trait NA made the model more accurate but trait NA itself did not contribute significantly to the model.

#### 3.4.2 State Negative Affect (H7)

By adding state NA to the LMM, it explained 64.6% of the total variance (χ^2^(24) = 3151.233, *p* < 0.001, Conditional R^2^ = 0.646), with the fixed effects explaining 12.5% of the variance (χ^2^(21) = 216.101, *p* < 0.001, Marginal R^2^ = 0.125.), 8.2% more than in the original model. The ANOVA Fixed Effect Omnibus tests showed that all the original predictors and the newly added state NA has a significant main effect: elbow joint position (F(4, 3282.5) = 7.10, *p* < 0.001, ηp^2^ = 0.009), stimulation site (F(3, 3272.9) = 4.19, *p* = 0.006, ηp^2^ = 0.004), trial number (F(1, 62.8) = 53.60, *p* < 0.001, ηp^2^ = 0.460), and state NA (F(1, 56) = 9.55, *p* = 0.003, ηp^2^ = 0.146). The elbow joint position * stimulation site interaction (F(12, 3282.7) = 10.51, *p* < 0.001, ηp^2^ = 0.037) also remained significant.

**FIGURE 5:**
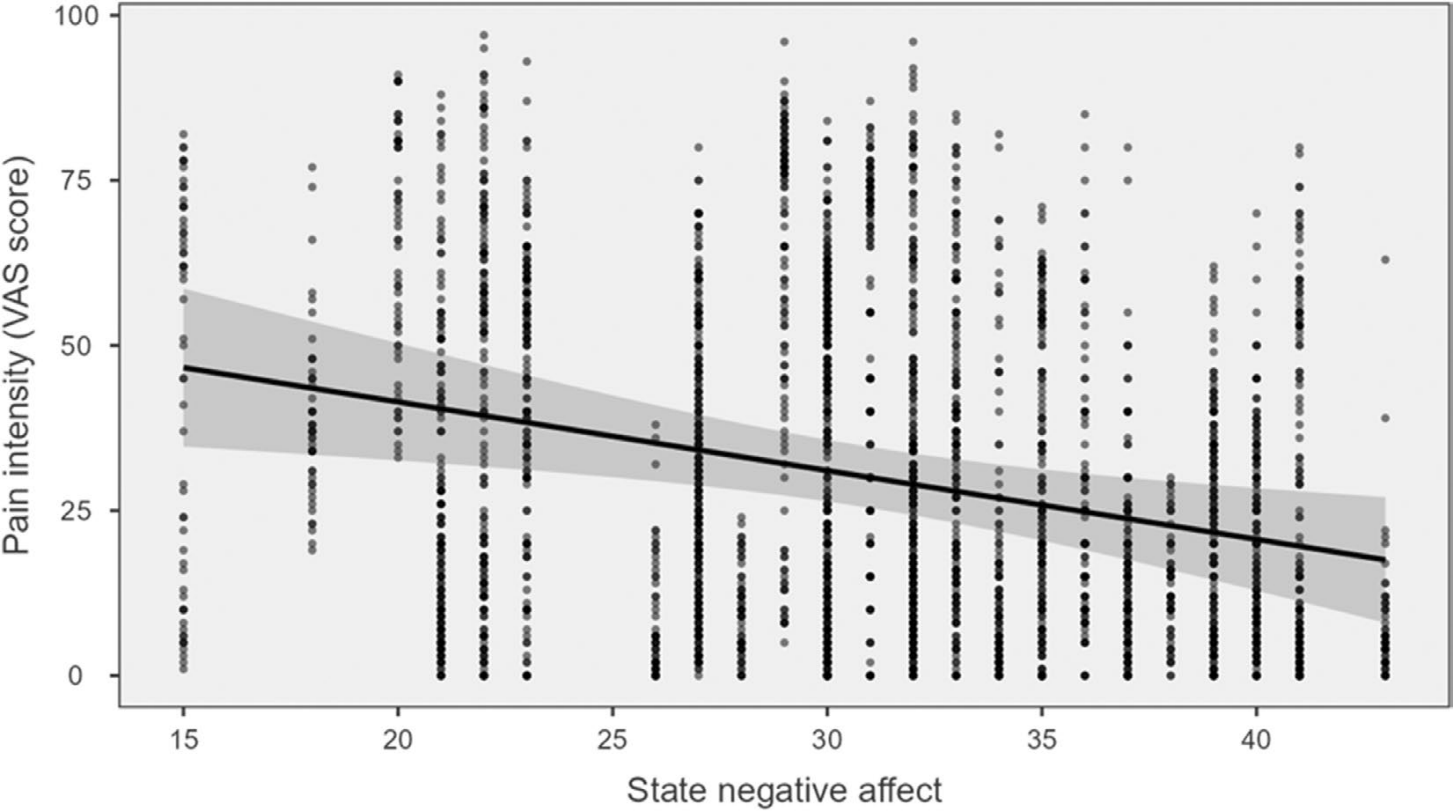
Partial correlation of pain intensity and state negative affect.

To understand the main effect of state NA we conducted a partial correlation analysis between state NA and pain intensity, while controlling for trial number, elbow joint position, and stimulation site, which showed a weak, negative correlation (rho = −0.279, *p* < 0.001), visualized in Figure 5.

#### 3.4.3 Somatosensory Amplification (H6)

By adding SSA to the LMM, it explained 64.6% of the total variance (χ^2^(24) = 3145.134, *p* < 0.001, Conditional *R* ^2^ = 0.646), with the fixed effects explaining 7.1% of the variance (χ^2^(21) = 210.002, *p* < 0.001, Marginal R^2^ = 0.07 1.), 2.8% more than in the original model. The ANOVA Fixed Effect Omnibus tests showed, that while all the original predictors remained significant—elbow joint position (F(4, 3282.4) = 7.11, *p* < 0.001, ηp^2^ = 0.009), stimulation site (F(3, 3272.9) = 4.19, *p* = 0.006, ηp^2^ = 0.004), and trial number (F(1, 62.8) = 53.66, *p* < 0.001, ηp^2^ = 0.461)—the newly added SSA (F(1, 56) = 2.98, *p* = 0.090, ηp^2^ = 0.051) did not show a significant main effect. The elbow joint position * stimulation site interaction (F(12, 3282.6) = 10.51, *p* < 0.001, ηp^2^ = 0.037) remained significant as well.

In summary, controlling for SSA made the model more accurate, but SSA itself did not contribute significantly to the model.

#### 3.4.4 Pain Catastrophizing

By adding PC to the model, it explained 66.3% of the total variance (χ^2^(24) = 2393.729, *p* < 0.001, Conditional *R* ^2^ = 0.663), with the fixed effects explaining 20.8% of the variance (χ^2^(21) = 166.583, *p* < 0.001, Marginal *R* ^2^ = 0.208.), 16.5% more than in the original model. The ANOVA Fixed Effect Omnibus tests showed that all the original predictors and the newly added PC has a significant main effect: elbow joint position (F(4, 2395.2) = 8.65, *p* < 0.001, ηp^2^ = 0.014), stimulation site (F(3, 2387.8) = 2.84, *p* = 0.037, ηp^2^ = 0.004), trial number (F(1, 44.6) = 42.07, *p* < 0.001, ηp^2^ = 0.485), PC (F(1, 40) = 16.13, *p* < 0.001, ηp^2^ = 0.287). The elbow joint position * stimulation site interaction (F(12, 2395.2) = 6.71, *p* < 0.001, ηp^2^ = 0.033) remained significant as well.

**FIGURE 6:**
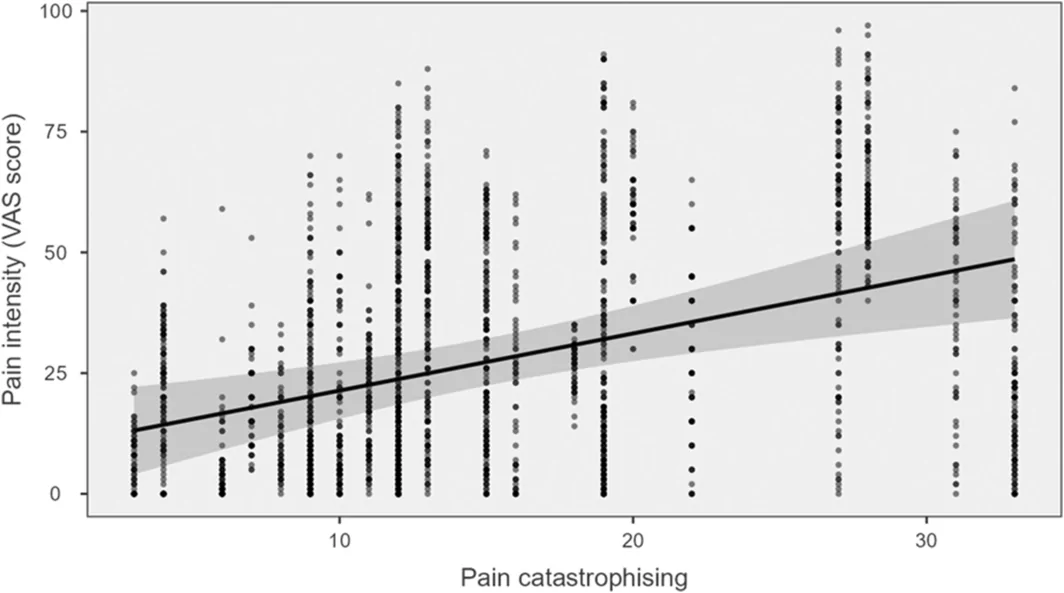
Partial correlation of pain intensity and pain catastrophizing.

To understand the main effect of PC, we conducted a partial correlation analysis between PC and pain intensity, while controlling for trial number, elbow joint position, and stimulation site, which showed a medium‐level, positive correlation (rho = 0.408, *p* < 0.001) visualized in Figure 6.

## 4 Discussion

In a laboratory experiment, perception of and skin‐conductance response to thermal pain stimulation administered on participants' forearms were influenced by the interplay between the angle of the elbow joint and the stimulation site along the proximal‐distal gradient. In addition, pain catastrophizing showed a positive association, while state negative affect showed a negative association with pain intensity, whereas no association was revealed with trait negative affect, somatosensory amplification and proprioceptive systematic error.

The major assumption of the present study was that the PPS is heterogeneous (Noel et al. 2021); we hypothesized that it shows a gradual attenuation from the midline of the body toward the periphery. As one of the major functions of the PPS is protecting the body from factors that pose a threat on its integrity (de Vignemont et al. 2021; Noel et al. 2021; Sambo and Iannetti 2013), we assumed that the pain reponse, including its physiological concomitants (SCR and HR, H2 and H3, respectively), evoked by nociceptive stimulation delivered on the forearm in different elbow joint positions (H1) and along a proximal‐distal gradient (H4) would show a decrease from center to the periphery. Our findings only partially supported our hypotheses. The stimulation site and elbow joint position showed a significant interaction, but the expected pattern did not emerge. If only the distance from the midline of the body mattered, pain intensities would increase with flexion, and these differences were more pronounced at distal stimulation sites, where the spatial distances from the body's midline are greater. In contrast, pain intensity at different elbow positions showed a different pattern at each stimulation site, with none of the patterns matching the expected pattern. In summary, H1‐H4 were not supported by the statistical analysis.

It is important to note that there are additional factors that might influence pain intensity, which might interact with the location of the stimulus in the PPS. Regarding the placement of thermal stimulation heads on the forearm, a proximal‐distal gradient in pain intensity was expected. The assumption that such a gradient exists was based on data on the density of small unmyelinated nerve fibers in the leg (McArthur et al. 1998). Apparently, this cannot be directly applied to the arms; for example, lower pain thresholds for the wrist region than for the elbow region were reported in a recent study (Masui and Asai 2024). Also, vibrotactile localization performance along the forearm showed a U‐shaped curve (Cholewiak and Collins 2003), which suggests that multiple factors might be at work here, leading to a pattern that is difficult to explain. Based on the main effect of stimulation site, s3 showed the lowest pain intensity, which does not support the existence of a proximal‐distal gradient. However, the disordinal nature of the interaction between stimulation site and elbow joint position questions the interpretability of the main effect. The same is true for the joint position main effect, where 120° showed the highest pain intensity. The interaction is also difficult to interpret. A possible explanation is that there might be other higher‐order effects at play in addition to the location within the PPS. Another mechanism might be related to the meaning of the body posture, as it can influence its emotional consequences (Phaf et al. 2014). Extending the elbows to 90° or more might place an individual in a more vulnerable body position, which might increase pain intensity. Another consideration is that the boundaries of PPS are dynamic, and depend on action‐capabilities (Brozzoli et al. 2010). Overly extending the arm may diminish the readiness for action, narrowing the PPS and changing the relative location of the stimulus within. Across the three more distal stimulation sites (from s2 to s4), high pain intensity of 120° might reflect the effect of a more vulnerable body posture, while the lower pain intensity in 150° might reflect the effect of further location within the PPS. The explanation for the most proximal stimulation site showing a different pattern remains up for debate. Altogether, our results imply that a complex interplay between proprioceptive information and stimulation site along the forearm influences pain intensity. Another possible confounding factor might be that different elbow joint positions are associated with different tonic discharge of proprioceptive receptors, which might also influence thermal pain perception. Further research is needed to distinguish these effects from each other.

In contrast to self‐reports, no effect on physiological concomitants of pain (HR and SCR; H3 and H4, respectively) was found. These null findings can be explained from different viewpoints. Firstly, it is possible that the responses were too weak and/or their variance was comparatively high, therefore the study was underpowered to detect them. Secondly, for SCR, which is consistently associated with nociceptive stimulation across multiple studies (Kyle and McNeil 2014), we found a significant habituation effect which could contribute to the lack of significant associations. Cardiovascular variables (HR, blood pressure), however, do not show a consistent response pattern (Kyle and McNeil 2014). In line with this finding, a recent systematic review showed that parasympathetic cardiac tone, as assessed via heart rate variability, is more consistently associated with pain intensity than sympathetic tone (Wegeberg et al. 2023). Thirdly, there is a well‐known discordance between physiological changes and their experiential counterparts (Köteles 2024); such a genuine dissociation can take place in the context of pain (Kyle and McNeil 2014). Finally, the position of the arms and the stimulation sites were in constant change during the experiment. This might have an impact on participants' attention, including the physiological concomitants of attentional shifts, which might have interacted with the changes caused by the nociceptive stimulation.

Contrary to our expectation (H5), the systematic bias in the accuracy of the perception of the position of the elbow joint was not associated with pain intensity. In other words, people prone to perceive their elbow joint more flexed than it actually is were not characterized by higher pain intensity in comparison to those with a bias toward extension (it is important to remember that measurements were conducted with covered eyes, that is, participants had to rely on proprioceptive cues in the determination of their forearm position). A possible explanation is that the variance of proprioceptive systematic error or its effect size was too small in our sample. Another possibility is that proprioceptive accuracy of the participants was reduced by repeated pain stimulation (Rossi et al. 1998), which could change their bias compared to the measurements taken before the experiment. Alternatively, the perception of joint position, and the proprioception‐based perception of pain in PPS share different mechanisms. In a similar vein, proprioceptive perception can dissociate from motor action or from the perception of body ownership (Gallagher et al. 2021; Platkiewicz and Hayward 2014).

Of the trait‐like characteristics associated with pain perception in the literature, SSA (H6) and trait NA (H7) were not related to pain intensity in our study. This is surprising as both constructs (that show a substantial overlap) are related to sensitivity to threats to the integration of the body (Köteles and Witthöft 2017) and a generally more defensive approach to perception, including the perception of bodily signals (Van den Bergh et al. 2021). On the other hand, pain catastrophizing, a construct that is more specifically related to pain perception than the former two, was positively related to pain intensity, that is, H8 was supported. Finally, state NA showed a negative association with pain intensity, which is surprising as a positive relationship was expected (H7). However, the relationship between negative affect and pain is not necessarily straightforward. In some cases, it might reduce pain, based on mechanisms like endogenous opioid release, changes in blood pressure, and distraction of attention (Janssen 2002). Alternatively, it is possible that participants with a more negative mood state, expected more intense pain before the stimulation period; therefore, pain was comparatively less intense than expected, which led to reporting lower pain intensity. Our explanation is admittedly speculative; a conceptual replication with multiple measurements of state negative affect would be necessary to better understand this counterintuitive finding.

From a broader perspective, this study was designed to shed more light on the assumed role of proprioceptive information in the construction/perception of the PPS. Proprioceptive systematic error did not influence pain intensity; however, as participants' eyes were closed during the pain stimulation, the effect of joint angle was based on proprioceptive information. The results, showing that the interaction of joint angle and stimulation site influences pain intensity, suggest that a more complex approach to PPS would be necessary (Cléry and Hamed 2021; Dijkerman and Medendorp 2021; Reed and Park 2021). Considering the fact that integration of sensory information is a genuine characteristic of the human brain (Pasqualotto et al. 2016; Stein and Stanford 2008), the exclusion of a modality that provides the central nervous system with information about the actual position of the arms from this process would be illogical. In addition, the PPS must show a strong association with the motor system in order to perform its basic functions (Noel et al. 2021), and it is well‐known that the regulation of the motor system relies partly on feedback (sensory‐based) and feedforward (expectation‐based) mechanisms (Seidler et al. 2004). Therefore, based on both theoretical considerations and empirical findings, we propose an extended definition of PPS, which includes the proprioceptive sensory modality in addition to the tactile, visual, and auditory modalities.

The major limitation of the present experiment concerns its generalizability (ecological validity): participants were young individuals, and the experimental setting was demanding and at the same time unusual to participants. Conceptual replications that focus only on one aspect (i.e., elbow joint angle or the proximal‐distal gradient) would represent fewer demanding conditions with possibly more straightforward conclusions. Also, 60 pain signals were delivered in total, which led to habituation. In addition, because of security considerations (i.e., we wanted to avoid tissue damage), all participants received identical thermal pain stimulation, which would have been better to be adjusted to their pain tolerance. Although we used a within‐subject design, it is possible that floor/ceiling effects reduced the variance of pain intensity ratings for certain individuals. Another limitation might be related to handedness. In this study, we assessed the right hand of every participant. Another approach would be to choose based on hand dominance (e.g., assessing the dominant hand). However, our approach is unlikely to influence results, as no difference between dominant and subdominant sites was found for this (ipsilateral) version of the JPR test (Goble and Brown 2007; Horváth et al. 2024).

### 4.1 Future Directions

One of the study's strengths is that it is among the first to thoroughly and systematically examine the phenomenon; however, as the article presents the results of a single study, a conceptual replication may be necessary from a basic research point of view. This is further supported by the difficulty in precisely explaining some of the results (i.e., the complex interaction pattern between the stimulation site and elbow joint position when pain intensity served as the outcome variable). Replications using different stimulation sites and including different body parts would be necessary to draw more definitive conclusions (e.g., testing how proximity to the body influences pain intensity when the stimulation is applied on the skin surface of the thigh). It would also be worthwhile to explore whether a similar pattern can be observed with different types of pain induction methods (i.e., electric, hypoxic, or cold). Regarding the proprioceptive aspect, the current task utilized a static joint position. It would be valuable to explore whether pain intensity is impacted by dynamic movement of the stimulus—approaching or moving away from the body. Based on how affective evaluation is influenced by motion (for a summary, see Horváth et al. 2025), not only the afferent signals, but the contextual meaning of the movement can be important (e.g., explicitly describing the situation as the source of pain approaching the participant). In terms of better understanding the PPS, another valuable line of research would be to test if other types of physical stimuli get intensified closer to the body. For example, it would be worthwhile to conduct similar experiments on affective touch, which is a positive stimulus in contrast to pain. In addition to behavioral aspects, the neural basis of the integration of nociceptive, proprioceptive, and visual signals merits further exploration. A more practice‐based research direction would be to test in medical situations whether pain can be reduced by placing the painful extremity further from the body midline.

## 5 Conclusion

The findings of the study suggest that, beyond the traditionally included tactile, visual, and auditory modalities, proprioception should be taken into consideration in a more complex conceptualization of peripersonal space. Also, our findings indicate that the hypothesized center‐to‐periphery gradient within the PPS is not affecting pain perception when only proprioceptive clues are given.

## Author Contributions

**Béla Bucsai:** data curation, formal analysis, methodology, investigation, writing – original draft, visualization, software, funding acquisition. **Áron Horváth:** writing – review and editing, formal analysis. **Ferenc Köteles:** conceptualization, project administration, resources, supervision, funding acquisition. **Ádám Koncz:** writing – review and editing, validation.

## Conflicts of Interest

The authors declare no conflicts of interest.

## Supporting information


**Table S1:** Model comparison of linear, logarithmic, and quadratic fit for habituation.
**Figure S1:** Effect of habituation on subjective pain experience.

## Data Availability

The data that support the findings of this study are openly available in Pain perception, proprioceptive accuracy and distance from the center of the body at https://osf.io/8x9y7/?view_only=a4ed5c09f49a4ffcbc954af87118f5c7.
