# Supplementary material for: Location of Nociceptive Stimulus in the Peripersonal Space Impacts Pain Experience
Source: Psychophysiology. 2026 Sep 5;63(9):e70396. doi: 10.1111/psyp.70396 (PMC13546002; doi:10.1111/psyp.70396)
Supplement: Supplementary file 1 [file PSYP-63-e70396-s001.docx]

Supplementary Material for

**Location of nociceptive stimulus in the peripersonal space impacts pain experience**

Béla Bucsai, Áron Horváth, Ádám Koncz, Ferenc Köteles

**Habituation trends**

To investigate the possible effect of habituation in our experiment we evaluated three different models for habituation. The first model assumed a linear, the second model a logarithmic, and the third a quadratic habituation trend. As anticipated, due to presenting numerous stimuli during a relatively short timeframe, we found a significant habituation to pain stimuli with all three models.

When comparing the models based on R², AIC, and BIC values (see Table S1) we found that the logarithmic fit (b = -3.85, SE = 0.283, t(3482.12) = -13.6, p = < 0.001) showed the best fit.

**Table S1.** Model comparison of linear, logarithmic, and quadratic fit for habituation

| Model | R² marginal | R² conditional | AIC | BIC |
| --- | --- | --- | --- | --- |
| Logarithmic fit | 0.020 | 0.616 | 29457.25 | 29481.94 |
| Quadratic fit | 0.019 | 0.615 | 29466.69 | 29497.55 |
| Linear fit | 0.015 | 0.611 | 29504.44 | 29529.13 |

Based on the results we decided to use logarithmically transformed version of the trial number to control for habituation in the main analyses. The overall effect of habituation is displayed on Fig S1.

**Fig S1.** Effect of habituation on subjective pain experience
